# No strings attached: new insights into epithelial morphogenesis

**DOI:** 10.1186/1741-7007-10-105

**Published:** 2012-12-20

**Authors:** Lance A Davidson

**Affiliations:** 1 3501 Fifth Avenue, 5059 BST3, University of Pittsburgh, Pittsburgh, PA 15213, USA

## Abstract

The dramatic ingression of tissue sheets that accompanies many morphogenetic processes, most notably gastrulation, has been largely attributed to contractile circum-apical actomyosin 'purse-strings' in the infolding cells. Recent studies, however, including one in *BMC Biology*, expose mechanisms that rely less on actomyosin contractility of purse-string bundles and more on dynamics in the global cortical actomyosin network of the cells. These studies illustrate how punctuated actomyosin contractions and flow of these networks can remodel both epithelial and planarly organized mesenchymal sheets.

## Commentary

**Figure 1:**
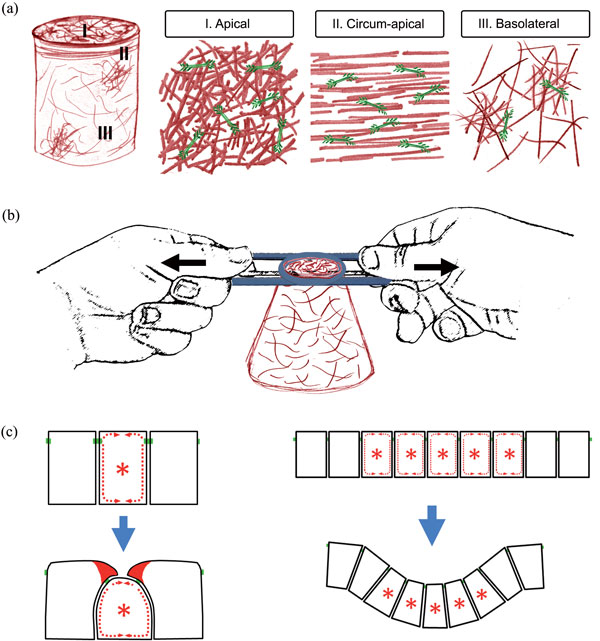
**Actomyosin contractility and flow, as well as neighboring cell extensions, shape epithelial sheets during morphogenesis**. **(a) **Actomyosin dynamics are not limited to these circum-apical bundles but are also found within the apical and basolateral cell cortex. **(b) **Classical 'purse-string' constriction draws circum-apical bundles of F-actin closed in a way analogous to the closure of a purse or noose. A constricting cell changes from cuboidal or columnar to adopt a wedge shape. Such a movement may concentrate proteins in the apical cortex or necessitate their removal by endocytosis. **(c) **Jacobson and colleagues hypothesized in their cortical tractor model that a flow of actomyosin over junctional adhesions may reshape neighboring cells, leading to cell wedging and folding. In the case of a single cell a cortical tractor (asterisk) could result in ingression; but when a field of cells (marked by asterisks) engages in tractor-tread like flows the entire sheet may fold.

Cell and developmental biologists over the past century have coined terms such as constriction, delamination, ingression, involution, and invagination to describe the diverse range of processes that shape epithelia globally into the body plan and locally into organ rudiments. In most cases these processes are accompanied by the narrowing of the apical cell cortex. When this cellular process appears autonomously in the manner of a closing 'purse-string' it is called apical constriction. Innovative genetic screens in *Drosophila*, *Caenorhabditis elegans *and mouse, as well as advances in light and electron microscopy, have revealed pathways that pattern and control these model cases of apical constriction during ventral furrow formation and dorsal closure in the fruit fly, as well as in the neural plate of vertebrates. In these and other cases of epithelial morphogenesis apical constriction appears so frequently that each iteration reminds one of a repeat production of the same stage play with nearly identical characters, props, and plotlines: the smoking gun of F-actin bundled into circum-apical rings, the leading role played by constricting cells, and a script consisting of autonomous programs of actomyosin contraction that draw a cell's neighbors toward each other as the purse-string of bundled actin contracts (Figure 1a). Recent papers, however, including one from Pohl *et al*. in *BMC Biology *on ingression during gastrulation in *C. elegans *[1], are introducing new elements into the play of epithelial morphogenesis, and questioning the role of the smoking gun of circum-apical actin in the process.

Actomyosin is both necessary and sufficient to drive apical constriction, so why 'smoking gun'? As initially formulated, the purse-string model was best represented by tissues isolated from adult retinal pigmented epithelia (RPE) [2]. The cells in the RPE are bound through adherens and tight junctions by dense, circum-apical bundles of actomyosin. When the cell sheet is gently permeabilized and exposed to calcium, myosins are activated, the bundles shorten, cells constrict their apices, and the sheet quickly bends. Since the cytoarchitecture of most epithelial sheets is defined by circum-apical bundles of actomyosin, this basic model of purse-string constriction has dominated thinking about processes that bend or shape these tissues. However, recent studies of morphogenesis in *Drosophila *and *Xenopus *have shown that epithelia and other planar cell sheets can be shaped not by actomyosin contraction of circum-apical bundles at the apical cell junctions, but by actomyosin contraction or actomyosin flow acting within the apical or basolateral cell cortex (Figure 1b) [3]. Elegant biophysical studies using laser ablation have even made it possible to characterize the relative contribution of actomyosin within the apical cortex and actomyosin with circum-apical bundles to tension in the *Drosophila *epithelium, and show how the contribution of the apical cortex increases with maturation of the embryo [4]. Many studies combining imaging and theoretical analysis (for example, [5]) demonstrate that differentially localized actomyosin arrays can serve as robust motors for epithelial morphogenesis; but little is known about the processes that control the frequency of their contractions, regulate their localization, or direct their assembly and disassembly.

## The importance of ensemble playing

Several recent papers highlight ways in which adhesion and the geometry of cells in tissues can convert the highly localized contractions of actomyosin arrays into cell- and tissue-shaping processes. Goldstein and co-workers [6] have shown that it is not the frequency of actomyosin contractions but how these contractions couple neighboring cells through cell junctions that dictate their efficiency in transmitting tension and directing cell shape changes. The nature of these couplings somehow dictates transmission of force from deeply positioned septate junctions between adjacent cells to the plane of the apical cortex. Since epithelial cells in *Drosophila *and *C. elegans *are under constant tension, the transmission or relaxation of tension plays a critical role in directing bulk movements such as germ band elongation and dorsal closure in *Drosophila *and during gastrulation and ventral enclosure in *C. elegans*.

Supracellular actomyosin bundles at the margins of tissues can play a comparable role in organizing cortical actin dynamics. Supracellular bundles of contractile actin bundles are commonly found at the margins of wounded epithelial sheets and at the boundaries between germ layers. One prominent bundle is found at the leading edge of the enveloping layer as the layer engulfs the yolk cell during zebrafish gastrulation. Recent studies of epiboly [7], a movement that stretches an epithelial sheet to completely enclose the embryo early during gastrulation, showed that significant tension exists within the cortex of the yolk cell perpendicular to the supracellular actin bundles at the leading edge of the enveloping cell layer. Tension in the yolk cell cortex correlates directly with the direction of actomyosin network flow in the cortex in a manner similar to the cortical flow seen in the apical cortex of actively contracting epithelial cells. The persistence of these forces in driving epiboly even when embryos are 'cylindrical' suggests these forces are frictional in nature rather than simply tensional. Frictional forces, from the flow of underlying actomyosin cortex in the yolk, are thought to pull on the bundled actin in the enveloping layer, drawing the enveloping layer toward the posterior end of the yolk ball. Interestingly, such a mechanism, termed the cortical tractor model, was first hypothesized in the 1980s by amphibian embryologist Antone Jacobson [8] to account for epithelial folding during neurulation (Figure 1c). Folding of the brain in amphibians involves progressive apical constriction coordinated with changes in cell height. Since many cells contribute to the folding, each cell need only adopt a small change in shape. Collectively, small forces could be summed across a broad field of cortically tractoring cells to produce a fold. Further investigations combining predictive models and biophysical experiments might be able to test the role of cortical flows in driving ingression or exclusion of individual cells from tightly integrated epithelial sheets [9].

## A little friction but no strings

In most of the cases discussed above the forces of contractile actin bundles in circum-apical junctions, in the apical cortex, or from frictional flows of actomyosin, are directed against strong attachment points - for example, as circum-apical belts of actin draw in neighboring cells at sites of cell-cell adhesion or as supercellular actomyosin structures at the margin of a wound or germ layer pull the layer over adjacent tissues. Attachment of the cortex to these fixed points is critical if forces generated by one cell are to be summed with those generated by others and transmitted across a tissue. The transmission of such forces coordinates large scale tissue movements that are necessary either to re-shape the tissue in a plane (as in convergent extension) or to direct out-of-plane bending moments that drive folding (for example, in neurulation). However, not all tissues are connected by these types of structures - important examples are mesenchymal tissues and less strongly polarized epithelial sheets - and these tissues also need to transmit force and coordinate large-scale movements.

**Figure 2:**
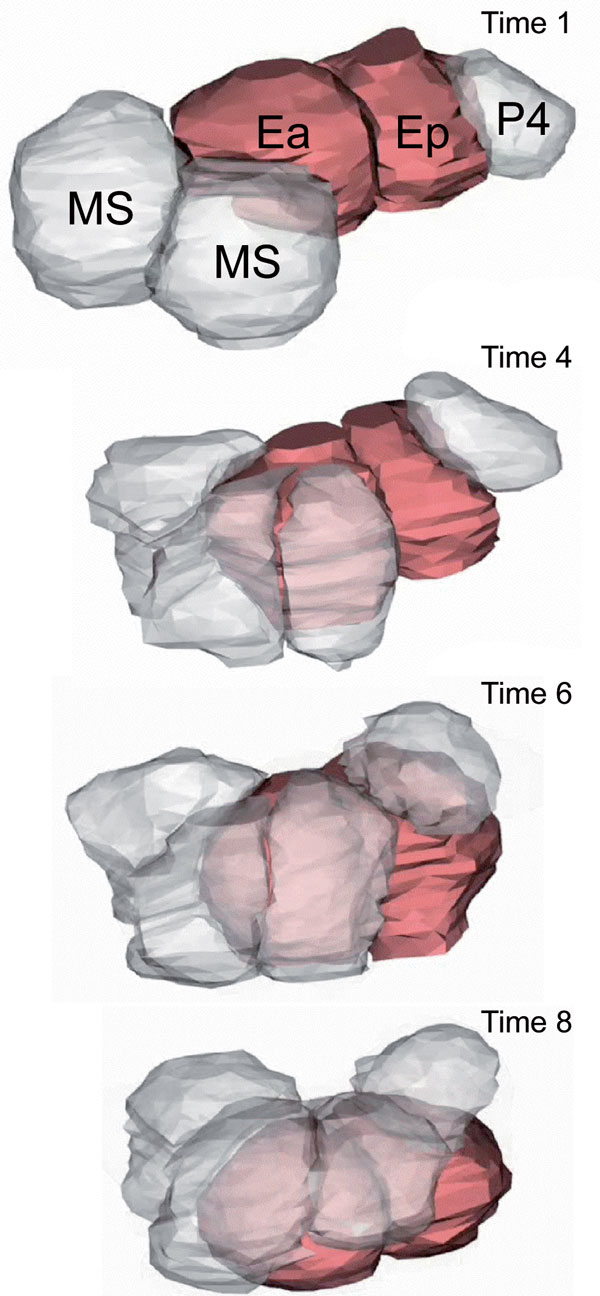
**Apical constriction, lateral cell extension, and ingression of endoderm cells**. Apical cell constriction with actomyosin flow in prospective endoderm cells Ea and Ep are complemented by cell protrusions extended from neighboring cells MS and P4 in Pohl *et al*. The combined effect of apical-directed flow and the engulfment of protrusions from neighboring cells removes the constricting cell from the surface, leaving in place a rosette formed by neighboring cells.

The internalization, or ingression of cells during *C. elegans *gastrulation described in *BMC Biology *by Pohl *et al*. illustrates such a case (Figure 2). Cells across the surface of the early *C. elegans *embryo move from the surface to the interior without discernible circum-apical bundles of actomyosin. Instead, these cells appear to trigger cell extensions in their neighboring cells. These extensions interact with pulses of actomyosin contractions within the diving cells to isolate these cells rapidly from the surface, moving them to the embryo's interior. The frequency and spatial organization of punctuated contractions and flow of actomyosin in the apical cell cortex during ingression is remarkably similar to events described in more tightly coupled epithelia and in loose mesenchymal cells engaged in morphogenesis, and parallels the model postulated by Jacobson 30 years ago. Interestingly, ingression appears to be an easily triggered emergent phenomenon, as the authors can drive cell internalization ectopically. Once the ingressing cells have been removed from the surface their former neighbors gather into rosette structures that rearrange the surface cell layer in preparation for further steps of embryogenesis. The internalization movements in *C. elegans *and the multicellular rosettes they leave behind appear remarkably similar to descriptions of cell ingression and cell extrusion during both later developmental events and tissue homeostasis.

The single-cell movements of ingression, intercalation, and extrusion share many features with coordinated epithelial movements that shape large cell sheets. Study of these events in controlled mechanical environments [10], as well as in complex mechanical environments such as the *C. elegans *embryo, hold great promise for understanding how similar molecular dynamics in the cytoskeleton control a diverse set of cell and tissue movements and bring us closer to understanding how normal development works and how dysregulation might lead to birth defects.
